# Antibody isotype analysis of malaria-nematode co-infection: problems and solutions associated with cross-reactivity

**DOI:** 10.1186/1471-2172-11-6

**Published:** 2010-02-17

**Authors:** Karen J Fairlie-Clarke, Tracey J Lamb, Jean Langhorne, Andrea L Graham, Judith E Allen

**Affiliations:** 1 Institutes of Evolution, Immunology and Infection Research, School of Biological Sciences, King's Buildings, University of Edinburgh, West Mains Road, Edinburgh, EH9 3JT, UK; 2 Current address: School of Biological Sciences, The University of Reading, Reading, Berks RG6 6UB, UK; 3 Division of Parasitology, National Institute for Medical Research, The Ridgeway Mill Hill, NW7 1AA, UK; 4 Department of Ecology and Evolutionary Biology, Princeton University, Princeton, NJ 08544, USA

## Abstract

**Background:**

Antibody isotype responses can be useful as indicators of immune bias during infection. In studies of parasite co-infection however, interpretation of immune bias is complicated by the occurrence of cross-reactive antibodies. To confidently attribute shifts in immune bias to the presence of a co-infecting parasite, we suggest practical approaches to account for antibody cross-reactivity. The potential for cross-reactive antibodies to influence disease outcome is also discussed.

**Results:**

Utilising two murine models of malaria-helminth co-infection we analysed antibody responses of mice singly- or co-infected with *Plasmodium chabaudi chabaudi *and *Nippostrongylus brasiliensis *or *Litomosoides sigmodontis*. We observed cross-reactive antibody responses that recognised antigens from both pathogens irrespective of whether crude parasite antigen preparations or purified recombinant proteins were used in ELISA. These responses were not apparent in control mice. The relative strength of cross-reactive versus antigen-specific responses was determined by calculating antibody titre. In addition, we analysed antibody binding to periodate-treated antigens, to distinguish responses targeted to protein versus carbohydrate moieties. Periodate treatment affected both antigen-specific and cross-reactive responses. For example, malaria-induced cross-reactive IgG1 responses were found to target the carbohydrate component of the helminth antigen, as they were not detected following periodate treatment. Interestingly, periodate treatment of recombinant malaria antigen Merozoite Surface Protein-1_19 _(MSP-1_19_) resulted in increased detection of antigen-specific IgG2a responses in malaria-infected mice. This suggests that glycosylation may have been masking protein epitopes and that periodate-treated MSP-1_19 _may more closely reflect the natural non-glycosylated antigen seen during infection.

**Conclusions:**

In order to utilize antibody isotypes as a measure of immune bias during co-infection studies, it is important to dissect antigen-specific from cross-reactive antibody responses. Calculating antibody titre, rather than using a single dilution of serum, as a measure of the relative strength of the response, largely accomplished this. Elimination of the carbohydrate moiety of an antigen that can often be the target of cross-reactive antibodies also proved useful.

## Background

The geographical and socio-economic distribution of malaria overlaps with areas in which a number of helminth parasites are also endemic. It is the norm in these areas for co-infection to occur and a growing body of literature reflects this [1-12]. The influence of co-infection on the immune response may result in either exacerbation or amelioration of disease [13-15]. It is therefore crucial to understand the host-parasite relationship in the context of multiple infections, if vaccine design and drug administration programmes are to be managed effectively [16]. Animal models accurately reflect many pathological aspects of malaria-helminth co-infection with regard to impact on disease outcome and also provide the opportunity to further examine immunological mechanisms in detail [17-20].

We previously undertook an investigation to assess the impact of a pre-existing chronic nematode infection on malaria-related pathology, utilising the rodent malaria *Plasmodium chabaudi chabaudi (Pcc) *and the rodent filarial nematode *Litomosoides sigmodontis (Ls) *[21]. We found that co-infected mice (*Pcc-Ls*), particularly those that did not have blood microfilaremia, had exacerbated immunopathology. This was associated with increased interferon-gamma (IFN-γ) responsiveness but was independent of *Pcc *parasitemia [21]. One of the primary objectives in our previous malaria-nematode co-infection studies was to gather antigen-specific T-cell data to determine whether nematode infection could alter the cytokine bias of the *Pcc*-specific T lymphocyte response towards Th1 and conversely, whether a potent Th1 response could alter the Th2 bias of the nematode-specific response.

Cytokine production by antigen specific T-cells can be difficult to assess during malaria, due to immune suppression associated with the peak of infection and apoptosis of splenocytes [22]. Additionally, the complex nature of the target antigen (*Pcc*-infected red blood cells) is a further complicating factor. Thus, gathering antigen-specific T-cell data remains a technical challenge of studying immunity to malaria particularly in human studies where there is the additional challenge of obtaining and maintaining lymphocytes in the field.

Here we focus on the dissection and interpretation of parasite antigen-specific antibody responses as an alternative to T-cell analysis. Antibodies of the IgG2a isotype are mainly produced by B cells in response to IFN-γ in mice [23-25] whereas the Th2 cytokine IL-4 switches B cells to produce IgG1 [24,26]. Although the generation of IgG1 as a marker for Th2 cells is less definitive than IgG2a as a marker of a Th1-type response, the ratio of IgG1 to IgG2a provides a powerful indicator of immune bias [27-30]. Measurement of antibodies can also be achieved with smaller sample volumes and poses fewer technical challenges than T-cell recall assays. Furthermore, antibody analysis can provide information on the fuller history of infection as it reflects cumulative immunological activity, whereas cytokine responses of T-cells are an *ex-vivo *'snapshot' that can more readily be altered by changes in the timing of sampling both *in vivo *and *in vitro*. Antibody analyses of co-infected animals might therefore provide evidence of overall Th1-Th2 cell cross-regulation even when cytokine analyses may not.

In addition to their use as indicators of cytokine bias during infection, antibody isotypes have direct functional relevance to disease severity in helminth-malaria co-infection. Antibodies are absolutely required for the ultimate clearance of malaria parasites [31]. In mice, antibodies of the cytophilic isotype IgG2a have been shown to recognise infected erythrocytes [32] and facilitate their destruction by phagocytes [33]. Similarly, in humans IgG1 and IgG3 are associated with enhanced parasite clearance [34]. If helminth co-infection alters antibody class-switching and consequently the production of malaria-specific cytophilic antibodies then the resolution of malaria infection may be affected. Indeed, co-infection with the gastro-intestinal nematode *Heligomosoides polygyrus *reduced *Pcc-*specific IgG2a responses and resulted in exacerbated malaria parasitemia [18]. There are also important implications for vaccine efficacy and administration. For example, immunisation that protected mice from malaria failed to do so in mice that also harboured a nematode infection [35].

In this study, the characterisation of antibody isotype responses as an indicator of cytokine bias during co-infection has proved unexpectedly challenging due to the production of cross-reactive antibodies induced by single-species infection. To establish the real effect of co-infection on the Th1/Th2 immune bias from non-specific reactivity to antigen we needed to determine how robust the cross-reactive responses were in comparison to the antigen-specific. We demonstrate that a combination of calculating antibody titre, from a dilution series of test sera, and periodate treatment of the parasite antigens can control for most cross-reactivity. The magnitude and robustness of some cross-reactivity, however merits further investigation to explore the potential function of these responses during co-infection.

## Methods

### Hosts, parasites and experimental infection

Specific pathogen free, 8-10 week old female BALB/c mice (Harlan, UK) were maintained in individually ventilated cages on diet 41b *ad lib *in a 12 h:12 h light-dark cycle. All experiments were carried out in accordance with the animals (Scientific Procedures) Act 1986, and were approved by the UK Home Office inspectorate and institutional review committee.

*Pcc *clone AS was originally isolated from thicket rats (*Thamnomys rutilans*) and was cloned by serial dilution and passage [36]. Parasites were recovered from frozen blood stabilates by passage through donor mice. Experimental parasite inoculations were prepared from donor mice by diluting blood in calf serum solution (50% heat-inactivated foetal calf serum, 50% Ringer's solution [27 mM KCl, 27 mM CaCl2, 0.15 M NaCl, 20 units heparin per mouse]). Each mouse received 0.1 ml of inoculum intraperitoneally (i.p) corresponding to an infective dose of 1 × 10^6 ^or 1 × 10^5 ^parasitized red blood cells (RBC), depending on the experiment. An inoculum of naïve RBC was given as a control for erythrocyte proteins.

The filarial nematode *Ls *was maintained by cyclical passage between gerbils (*Meriones unguiculatus*) and mites (*Ornithonyssus bacoti*) as described previously [37]. Infection was initiated by subcutaneous (s.c) injection of 25 infective (L3) larvae. For co-infection experiments in which the influence of malaria on chronic nematode infection was addressed, 1 × 10^6 ^*Pcc *parasitized RBC were introduced i.p on Day 60 of an established *Ls *infection and mice were sacrificed on day 20 post-*Pcc *infection, as described previously [21]. Whole blood was collected from the brachial artery and serum recovered after clotting at room temperature.

*Nb *worms were maintained by serial passage through Sprague-Dawley rats. L3 larvae were obtained by culturing the faeces of infected rats at 26°C for a minimum of 5 days [38]. For acute nematode-malaria co-infection, infection was initiated by s.c injection of 200 infective (L3) larvae on the same day that *Pcc *was introduced by inoculation i.p of 1 × 10^5 ^parasitized RBC. Mice were sacrificed on Day 20 post-infection under terminal anaesthesia. Whole blood was collected from the brachial artery and was separated using Sera Sieve (Hughes & Hughes Ltd).

### Antigens

Two malaria antigens were used in this study: a recombinant protein and a crude antigen homogenate prepared from parasitized erythrocytes. The recombinant Merozoite Surface Protein-1_19 _(MSP-1_19_) was originally sequenced, cloned and expressed from *Pcc *AS clone, as described previously [39]. In brief, the MSP-1_19 _nucleotide sequence was inserted into *Pichia pastoris *vector pIC9K and protein expression carried out in *Pichia pastoris *strain SMD1169. This antigen was used in ELISA at a concentration of 1 μg/ml.

The crude malaria homogenate - lysed *Pcc *parasitized red blood cell extract (pRBC) - was prepared from whole blood of mice with a parasitemia in excess of 20%. Mice were bled by cardiac puncture with a heparinised syringe and blood stored at -80°C prior to 3 rounds of freeze-thaw to lyse the parasitized red blood cells. The lysed cells were sonicated, on ice, twice for 30 sec at 10 Amp and centrifuged at 16060 g for 10 min. The supernatant was stored at -80°C. Similarly, a naïve red blood cell extract (nRBC) was prepared as a control for RBC proteins; responses to this antigen amongst infected mice were indistinguishable from naïve (data not shown). In the *Ls *experiments this antigen was used in ELISA at 0.5 μg/ml and in the *Nb *experiments at 5 μg/ml.

*Ls *and *Nb *extracts (LsA and NbA) were prepared by homogenisation of adult nematodes in PBS. The somatic extracts were centrifuged at 1000 g for 20 mins and the pellet discarded. The extract was stored at -20°C. LsA was used in ELISA at 0.5 μg/ml and NbA at 5 μg/ml.

### Antibody detection

ELISA was used to measure antigen-specific IgG antibodies in the serum of nematode-infected, *Pcc-*infected or co-infected mice. In the *Pcc-Ls *study, sera were added in a serial dilution 1/100 - 1/400 and a dilution was then chosen whereby all samples fell in the linear range of the curve; for IgG1, a dilution of 1/200 and for IgG2a 1/100. For the subsequent *Pcc-Nb *study, serum samples were added in a serial dilution 1/50 - 1/819200. Antibody titres were calculated as the reciprocal of the greatest dilution at which optical density (O.D) was greater than the mean plus 3 standard deviations of the O.D values observed for control mouse sera at 1/200 dilution.

Antibody responses to MSP-1_19_, pRBC, NbA or LsA were determined for IgG isotypes IgG1, IgG2a, and IgG3. 96 well maxisorp immunoplates (Nunc) were coated at 4°C overnight with either recombinant or crude antigens at the concentrations indicated (see Antigens section) in 0.06 M carbonate buffer (0.04 M NaHCO_3_, 0.02 M NaCO_3_, pH9.6) in a final volume of 50 μl per well. Non-specific binding was blocked with 5% FCS in carbonate buffer (200 μl/well) for 2 hours at 37°C. Wells were washed three times in Tris buffered saline with 0.1% Tween (TBST) after each step. Serum samples were added in serial dilutions as indicated using TBST as a diluent, in a final volume of 75 μl per well and incubated for 2 hours at 37°C. Isotype specific detection antibodies were diluted in TBST in a final volume of 50 μl per well. For IgG1, HRP conjugated goat anti-mouse IgG1 (Southern Biotech 1070-05) was used at 1/6000, HRP conjugated goat anti-mouse IgG2a (Southern Biotech 1080-05) at 1/4000 and HRP conjugated goat anti-mouse IgG3 (Southern Biotech 1100-05) was used at 1/1000. Plates were incubated for 1 hour at 37°C. An additional wash in distilled water was carried out before developing with ABTS peroxide substrate (Insight Biotechnology), 100 μl per well, at room temperature for 20 minutes. O.D was read at 405 nm using a spectrophotometer.

Polyclonal IgE levels were determined by sandwich ELISA. 96 well maxisorp immunoplates (Nunc) were coated overnight at 4°C with 100 μl of IgE capture antibody (2 μg/ml; clone R35-72 Pharmingen) diluted in carbonate buffer. Plates were blocked with 5% non-fat skimmed milk in carbonate buffer for 2 hr at 37°C. Plates were washed 5 × in TBST before addition of sera at 1/10 and 1/20 dilutions in a final volume of 50 μl/well and left overnight at 4°C. For the standard curve two- fold serial dilutions of purified mouse IgE, κ monoclonal isotype standard (Pharmingen) were used. After 5 washes in TBST, 100 μl of biotinylated detection antibody (2 μg/ml; clone R35-118 Pharmingen) diluted in TBST with 5% FCS was added and plates left at 37°C for 90 mins. Plates were washed 5 × in TBST prior to incubation with ExtrAvidin peroxidase (SIGMA), diluted 1:8000 in TBST with 0.5% FCS, for 30 mins at 37°C. After a final wash in distilled water, plates were developed with 100 μl TMB microwell peroxidase substrate system (Insight Biotechnology Ltd) and read at 650 nm.

In order to determine the extent to which carbohydrate or protein moieties contributed to the antibody response the antigens were pre-treated with periodate. Antigen-specific IgG1, IgG2a and IgG3 antibodies were measured in response to antigens treated with periodate. The ELISA was carried out as detailed for the *Nb *co-infection experiment with untreated antigens but the following additional steps were included after blocking with 5% FCS: carbonate buffer, prior to sample addition. TBST wash (×3) was followed by the addition of 10 mM sodium (meta) periodate diluted in 50 mM sodium acetate in a final volume of 100 μl/well. Plates were incubated at 37°C for 1 hour and then washed in 50 mM sodium acetate. To stop the activity of periodate, 100 μl of 50 mM sodium borohydride solution was added to each well.

### Statistical Analysis

General linear statistical models allowed us to frame and test questions such that we could determine whether differences in infection status and/or presence of carbohydrate antigen explained the observed variation in antibody responses. For more detailed explanation of the statistical methods employed see Grafen and Hails [40]. Infection status and treatment with periodate (or not) were included as categorical factors and their ability to predict antibody response was formally evaluated via Analysis of Variance (ANOVA). The serial dilution of sera in an ELISA produces ordinal data, which were log_10 _transformed prior to analysis to ensure the data were approximately normally distributed, in accordance with the requirements of linear models. Analyses were carried out using the statistical package JMP 5.1 (SAS). The maximal model was fitted first and minimal models were obtained by sequentially removing non-significant terms (P-value > 0.05), beginning with interactions. Finally, whenever a factor was significant (*P *< 0.05), an All Pairs Tukey post-hoc test was carried out to identify which groups of mice differed significantly in antibody induction, with respect to infection status or periodate treatment.

## Results

### Antibody isotype responses are skewed by malaria-filaria co-infection but cross-reactivity confounds data interpretation

We had previously used a model of *Pcc*-nematode co-infection in which *Pcc *is introduced into mice with pre-existing chronic *Ls *infection to investigate the dynamics of infection with regard to parasitological outcome and cytokine bias [21]. In the *Pcc-Ls *model the peak of malaria parasitemia was controlled by day 10 and resolved by day 14 post Pcc-infection. For this study, we envisaged that analysis of the antibody isotype response from these mice would provide a method to rapidly and quantitatively assess immune bias. We thus asked whether we could use antibody isotype ELISA for IgG2a and IgG1 to address whether nematode infection would skew the Th1 cell response to *Pcc *to a more Th2 cell biased response. Conversely, we wished to address whether the powerful Th1 cell response induced by malaria would have the capacity to alter an established IgG1 response to a nematode infection. As is common practice in many studies [41-48], especially with large sample sizes as in this study, the ELISAs were performed with a fixed serum concentration derived from the linear point in a dilution series.

**Figure 1:**
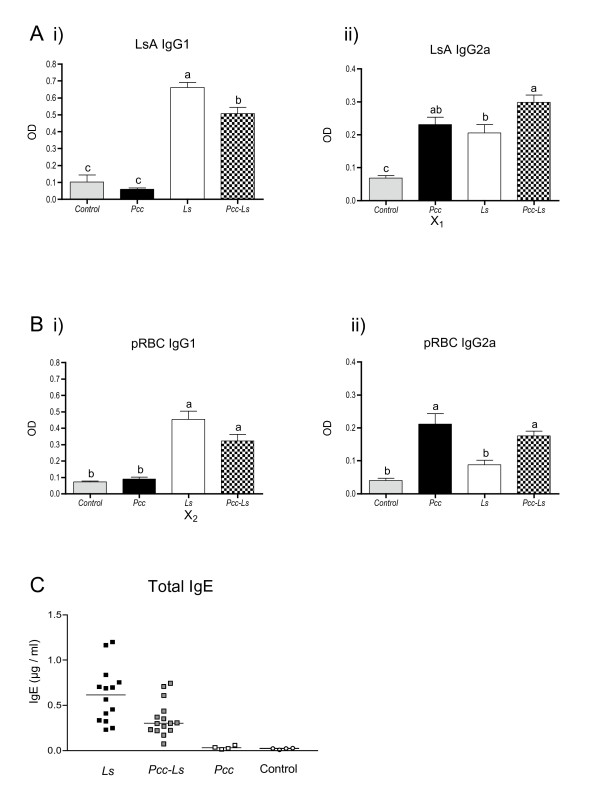
**Antibody isotype responses in infection and co-infection with *Litomosoides sigmodontis *and malaria**. Mice were infected with 25 *Ls *L3 larvae on day 0 and/or 10^6 ^*Pcc*-infected RBCs on day 60 post-*Ls *infection. Serum antibody responses measured by ELISA at day 80 post-*Ls *infection to (A) *Ls *antigen (LsA) and (B) crude *Pcc *infected RBC antigen (pRBC). Th2 isotype IgG1 is shown in (Ai) and (Bi) whilst the Th1 associated isotype IgG2a is shown in (Aii) and (Bii). Grey bars represent control mice, black bars represent *Pcc *mice, white bars the *Ls *mice and the chequered bars co-infected mice (*Pcc-Ls*). The letter X highlights those responses that are cross-reactive. Groups not connected by the same letter denote pairs that are significantly different according to Tukey's Pairwise analysis while those that share the same letter do not differ significantly. (C) Polyclonal IgE responses. Black squares represent *Ls *mice, white squares represent *Pcc *mice and grey squares represent co-infected (*Pcc-Ls*) mice. White circles indicate control animals. Data shown in A & B are compiled from 3 experiments (control mice n = 13, Ls mice n = 37, Pcc mice n = 14, Pcc-Ls mice n = 41).

As expected from previous studies [49-51], we were able to detect an IgG1 response against LsA in *Ls *mice. Co-infected (*Pcc-Ls*) mice also produced LsA-specific IgG1, but it was reduced in magnitude compared to the *Ls *mice (Fig 1Ai). Thus co-infection with *Pcc *appeared to down-regulate the anti-*Ls *specific IgG1 response. It also appeared that responses in *Pcc-Ls *mice were further biased toward a Th1 cell response through the induction of IgG2a to LsA (Fig 1Aii). Also as expected [32,52], *Pcc *mice mounted highly biased Th1-cell responses as indicated by the predominance of *Pcc *(pRBC)-specific IgG2a over IgG1 (Fig 1B). Once again, *Pcc-Ls *mice appeared to alter this bias by increasing the amount of pRBC-specific IgG1 in comparison to the *Pcc *mice (Fig 1Bi).

At first glance, the results strongly suggested that the isotype and hence cytokine bias of each single-species infection was significantly impacted by co-infection. In support of this, polyclonal IgE was highest in *Ls *mice and absent in *Pcc *mice with *Pcc-Ls *mice exhibiting an intermediate level (Fig. 1C) although *Pcc-Ls *mice did not differ statistically from *Ls *mice in this polyclonal analysis. However, it was apparent that *Pcc *mice were exhibiting sizable antibody responses to LsA (X_1 _in Fig 1Aii) and conversely *Ls *mice were exhibiting strong antibody responses to pRBC (X_2 _in Fig 1Bi). Western blot analysis confirmed that these *Ls*-induced cross-reactive responses were directed against the parasite rather than the RBC (data not shown). We thus had to ask whether the shift away from LsA-specific IgG1 toward IgG2a responses in the *Pcc-Ls *mice was due to the influence of IFN-γ on the *Ls*-induced response or simply reflected the presence of cross-reactive *Pcc-*induced IgG2a responses to LsA. Conversely, was the apparent increase in pRBC-specific IgG1 responses in *Pcc-Ls *mice due to *Ls-*induced antibodies that cross-reacted with infected red blood cells? Because serum titres had not been determined in this study, we could not assess the relative strength of cross-reactive versus antigen-specific responses. Our observation that there was significant cross-reactivity at the sera dilution tested thus confounded our ability to interpret any changes in immunological bias during *Pcc-Ls *co-infection.

### Cross-reactive antibody responses are also observed during malaria-*Nippostrongylus brasiliensis* co-infection

In order to address the utility of antibody isotype responses further, we embarked on co-infection experiments with *Pcc *and the nematode *Nb*. Because *Pc-Nb *is an acute model, whereby the nematode is cleared by day 7 and the peak of malaria parasitemia is controlled by day 10, the antibody data could be collected after only 20 days of co-infection, a more practical time frame than the 80 days required for the *Pcc-Ls *experiments. This also allowed us to address whether our observations of antibody cross-reactivity were a more general feature of *Pcc*-nematode infection. Given the apparent cross-reactivity observed at a fixed dilution of sera in the *Pcc-Ls *ELISA we used endpoint titres derived from a serial dilution (1:50 - 1:819200) in the *Pcc-Nb *assays to address whether this readout would overcome cross-reactivity problems. To determine if the specificity of the assay could be improved with the use of recombinant antigens we also included the malaria protein, MSP-1_19 _[39] not available to us for the *Ls *studies.

**Figure 2:**
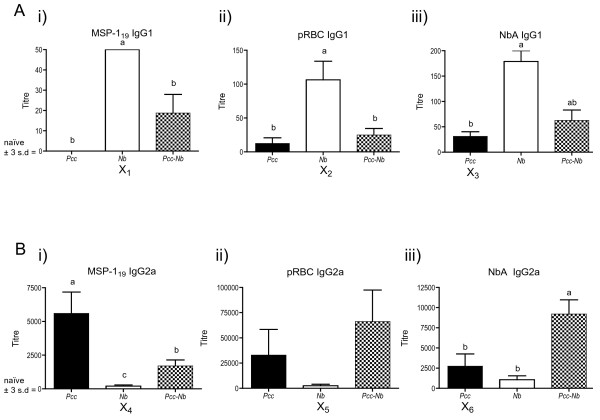
**Antibody isotype responses in infection and co-infection with *Nippostrongylus brasiliensis *and malaria**. Mice were infected with 200 *Nb *L3 larvae and/or 10^5 ^*Pcc*-infected RBCs on day 0. Serum antibody titres (A) IgG1 (B) IgG2a to recombinant *Pcc *antigen MSP-1_19 _(i), crude *Pcc *antigen (*pRBC*) (ii) and crude *Nb antigen *(*NbA*) (iii) were measured at day 20 post-infection for 8 mice per infection group. Black bars represent the *Pcc *mice, white bars the *Nb *mice and the chequered bars the co-infected mice (*Pcc-Nb*). Antibody titres are shown on the y-axis and represent the reciprocal of the greatest dilution at which O.D was greater than the mean plus 3 standard deviations of the O.D values observed for control mouse sera at a 1/200 dilution. The letter X highlights those responses that are cross-reactive. Groups not connected by the same letter denote pairs that are significantly different according to Tukey's Pairwise analysis.

The antibody responses we observed on Day 20 of *Pcc-Nb *co-infection (Fig 2) paralleled those we had seen in the *Pcc-Ls *experiments at Day 80. For example, as seen in Fig 2Aiii, *Nb *mice made IgG1 biased responses against NbA, and responses in *Pcc-Nb *mice were intermediate between *Nb *and *Pcc *mice. In addition, *Pcc *mice mounted a strong MSP-1_19_-specific IgG2a response that was reduced in *Pcc-Nb *mice (Fig 2Bi). As before, levels of polyclonal IgE in *Pcc-Nb *mice were intermediate (data not shown). We again observed cross-reactivity, whereby *Nb *mice mounted detectable IgG1 and IgG2a responses to both recombinant and crude malaria antigens (indicated by X_1&2 _in Fig 2A and X_4&5 _in Fig 2B, respectively). The magnitude of the *Nb*-induced IgG2a cross-reactive response is particularly striking with titres against crude and recombinant malaria antigens reaching 2500 and 200 respectively. Similarly, *Pcc *mice mounted responses to NbA (X_3 _in Fig 2A and X_6 _in Fig 2B). It is important to note that these titres although low are markedly greater than background responses (mean plus 3 standard deviations of serum responses from control mice), which are represented as zero on the y-axis. The immune bias that is apparent in serum antibody isotype responses is fully supported by cytokine responses in the lymph nodes of *Pcc-Nb *infected mice as we have recently described [53]. Of interest, no cross-reactivity was observed at the T-cell level.

### Cross-reactive IgG1 responses of malaria-infected mice to NbA are lost at higher dilutions but IgG2a responses remain

The analysis of both *Pcc-Ls *and *Pcc-Nb *co-infection indicates that the issue of cross-reactivity is a factor investigators are likely to routinely encounter. Determining the qualitative and quantitative aspects of the cross-reacting antibody responses are not only important for the practical analysis of immune deviation but could be of real biological relevance during co-infection.

As expected, antibody responses were biased, in terms of isotype, by infection status. The bias in isotype due to a particular infection (Th2 associated IgG1 induced during *Nb *infection, for example) was extended to non-specific antigens, as seen in the IgG1 response of *Nb *mice to both MSP-1_19 _and pRBC (X_1 _and X_2 _in Fig 2Ai + 2Aii). However, *Pcc*-specific IgG2a titres in *Pcc *mice were significantly higher than the cross-reactive response induced in *Nb *mice (Fig 2Bi). Thus, although *Nb *mice made cross-reactive IgG2a responses, these were no longer detectable with increasing dilution of sera (Fig 2Bi). In this case capitalising on the differences in strength of antigen-specific and cross-reactive responses clarified interpretation of immune bias in co-infected mice. Similarly, IgG1 responses to NbA were significantly higher in *Nb *mice than the cross-reactive response induced by *Pcc *mice (Fig 2Aiii). However, titre of *Pcc*-induced cross-reactive IgG2a to NbA did not differ significantly from *Nb *mice (X_6 _in Fig 2Biii).

We can conclude from this analysis that cross-reactive IgG1 responses to NbA were only detectable at dilutions less than 1:100 and thus higher dilutions may be used to avoid cross-reactivity when assessing antigen-specific antibody isotype profiles for the purpose of interpreting immune bias. However, increasing sera dilution did not always overcome the cross-reactivity observed, as IgG2a responses to NbA in *Pcc *mice were still observed at 1:2500. This cross-reactivity warrants further investigation, as it is likely to be important biologically. Indeed even cross-reactive responses detectable only at high serum concentrations may still have functional relevance *in vivo*.

### Cross-reactivity appears to lie predominantly with carbohydrate epitopes and can be largely eliminated by periodate treatment

**Figure 3:**
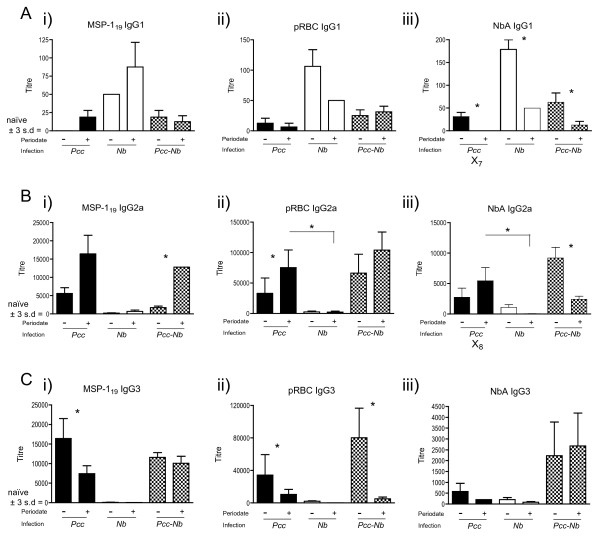
**Antibody responses to protein epitopes in infection and co-infection with *Nippostrongylus brasiliensis *and malaria**. The parasite antigens used in ELISA were pre-treated with periodate to disrupt carbohydrate epitopes, in order to determine if cross-reactive responses target the carbohydrate or protein moiety of an antigen. Mice were infected with 200 *Nb *L3 larvae and/or 10^5 ^*Pcc*-infected RBCs on day 0. Serum antibody titres (y-axis) were measured at day 20 post-infection for 8 mice per infection group and represent the reciprocal of the greatest dilution at which O.D was greater than the mean plus 3 standard deviations of the O.D values observed for control mouse sera at 1/200 dilution. Antibody isotypes (A) IgG1 (B) IgG2a (C) IgG3 to recombinant *Pcc *antigen MSP-1_19_ (i), crude *Pcc *antigen (*pRBC*) (ii) and crude *Nb antigen *(*NbA*) (iii) before (-) and after (+) treatment with periodate are shown. Black bars represent *Pcc *mice, white bars the *Nb *mice and the chequered bars the co-infected (*Pcc-Nb*) mice. (*) denotes pairs which are significantly different according to Tukey's pairwise analysis.

Antibody cross-reactivity in a broad range of systems can be attributed to reactivity with carbohydrate determinants [54,55]. Additionally, the IgG3 isotype is often associated with recognition of carbohydrates [56] and we observed cross-reactive IgG3 antibody responses during *Pcc-Nb *co-infection (Fig. 3). *Nb *mice mounted IgG3 responses to both MSP-1_19 _and pRBC antigens, achieving titres of 200 and 3200 respectively (Fig 3Ci and 3Cii). *Pcc *mice mounted similar cross-reactive IgG3 responses to NbA (Fig 3Ciii). We thus chose to assess whether cross-reactivity in our *Pcc-Nb *co-infection system could be overcome by periodate treatment of the parasite antigens. Periodate oxidises carbohydrate to aldehydes, thus disrupting carbohydrate epitopes, which allowed us to distinguish if cross-reactive responses target the carbohydrate or protein moiety of an antigen. This could be of particular importance where detection of cross-reactive responses was not overcome by increasing serum dilution. In addition to clarifying the interpretation of shifts in immune bias, determining whether induction of specific isotype responses is driven by protein or carbohydrate recognition has important implications for vaccine design and diagnostic serology.

For the pRBC and MSP-1_19 _antigens, periodate treatment did not significantly affect recognition by IgG1 antibodies (P ≥ 0.7). Periodate treatment of NbA however, significantly reduced anti-NbA IgG1 titres across all infection groups (Fig 3Aiii), suggesting common recognition of a carbohydrate moiety. In particular, the cross-reactive recognition of NbA, by IgG1 antibodies from *Pcc *mice, was ablated (X_7 _in Fig 3Aiii).

Treatment of MSP-1_19 _antigen with periodate resulted in a significant increase in IgG2a detected in sera from *Pcc-Nb *mice. Detection of IgG2a in singly-infected mice also followed this trend but was not statistically significant (Fig 3Bi). These results suggest recognition of a protein epitope on the recombinant antigen previously masked by glycosylation. *Plasmodium *species lack the glycosyltransferases required for any glycosylation other than attachment of GPI anchors [57,58]. However, inappropriate glycosylation of the recombinant protein can occur in the *Pichia *expression system [57]. The increase in protein-specific responses, following periodate treatment of the recombinant antigen (MSP-1_19_), may thus reflect the response to the natural non-glycosylated parasite protein seen during infection. IgG2a recognition of periodate treated pRBC antigen was enhanced in sera from *Pcc *mice, though not in the *Pcc-Nb *or *Nb *mice (Fig 3Bii). This suggests the cross-reactive IgG2a response is not targeting carbohydrates, which could potentially be conserved amongst parasite antigens.

For NbA, it was only after treatment with periodate that differences amongst infection groups become apparent, whereby significantly greater amounts of (cross-reactive) IgG2a antibodies were detected in *Pcc *mice in comparison to *Nb *mice (X_8 _in Fig 3Biii). Thus, the *Pcc*-induced cross-reactive IgG2a response to NbA (X_6 _in Fig 2Biii) that was not lost with serial dilution was also maintained following periodate treatment (X_8 _in Fig 3Biii). In contrast, the *Nb*-specific IgG2a response appears to target carbohydrate as periodate treatment reduced recognition of NbA in both *Nb *and *Pcc-Nb *mice (Fig 3Biii) although only statistically significant in the *Pcc-Nb *mice. This may indicate that atypical Th1-type responses to helminth antigens are driven by carbohydrate.

Disruption of carbohydrates via periodate treatment significantly affected the recognition of *Pcc *antigens by IgG3 antibodies. In particular, recognition of periodate treated pRBC was reduced in serum from all mice that had experienced *Pcc *infection; this was most evident in the *Pcc-Nb *mice (Fig 3Cii). The recognition of periodate treated MSP1-_19 _was also significantly reduced in *Pcc *mice (Fig 3Ci). The more pronounced reduction in IgG3 response to the treated pRBC antigen may reflect the greater proportion of carbohydrate components in this crude antigen preparation in comparison to the single recombinant protein.

## Discussion

Antibody analysis should be able to provide critical information on changes in cytokine bias due to co-infection. This is particularly important in human studies where serum may be the only reagent available for immunological analysis. Whilst we acknowledge that there is a need to confirm the relationship between splenic or serum cytokines and antibody responses in co-infection if this strategy is to be used in human studies, our focus is on the interpretation of antigen-specific Th1/Th2 bias based on antibody isotype, which was complicated by cross-reactivity in the two co-infection models studied here. It is worth noting that cross-reactive responses were observed regardless of whether recombinant or crude antigens were used. We primarily address technical strategies that will enable us, and others, to draw conclusions regarding the influence of a co-infecting parasite on immune bias using serum antibodies. However, the functional implications of cross-reactive responses are also discussed.

Murine models that aim to dissect the real effect of a co-infecting parasite on immune bias must use large numbers of animals to detect significant differences in antigen-specific responses between single and dual infection. Thus for antibody analysis of the large sample size (see legend Fig 1 for details) in our *Pcc-Ls *study of co-infection we chose a fixed serum concentration, previously determined to fall within the linear range of the dilution curve. Although this saved time and reagents, in retrospect, it provided insufficient information for our purposes: it did not allow us to distinguish the relative strengths of cross-reactive versus antigen-specific responses.

When antibody titres were calculated in the *Pcc-Nb *study, we were able to determine whether apparent alterations in antibody isotype profile on co-infection were due to actual changes in parasite-specific responses or reflected a cross-reactive response. For example, determining that cross-reactive IgG2a antibody titres in *Nb *mice (X_4 _in Fig 2Bi) were significantly lower than the antigen-specific response of *Pcc *mice meant that cross-reactivity was unlikely to influence the titre observed in *Pcc-Nb *mice. This allowed us to conclude that the reduction in Th1 type antibody in *Pcc-Nb *mice was probably due to suppression of *Pcc*-specific Th1 responses by nematode infection. Further to this, had we not calculated titre and relied on optical density data derived from a single dilution of sera we may not have observed the difference between *Pcc *and *Pcc-Nb *mice and thus incorrectly concluded that there was no effect of co-infection on Th1 responses. Similarly, analysis of antibody titre enabled us to detect the reduction in anti-NbA IgG1 antibody in *Pcc-Nb *mice (Fig 2Aiii), which suggests a *Pcc*-mediated bias toward a Th1 cell response. In other cases, cross-reactivity was observed even at high dilutions with *Pcc *mice achieving IgG2a titres equivalent to or greater than *Nb *mice (X_6 _in Fig 2Biii). In this case, calculation of titre did not help to unravel potential cytokine influences and the enhanced IgG2a response in co-infected mice may be due to increased Th1 cytokines during co-infection and/or the presence of cross-reactive antibody (Fig 2Biii).

Nematode surface antigens and the excretory/secretory products from these parasites are heavily glycosylated [59]. Similarly, *Plasmodium *species express glycoconjugates on their surface and have abundant glycophosphatidylinositol anchors [60]. In other co-infection systems cross-reactive epitopes have been shown to derive from carbohydrate structures [61]. The sensitivity of the carbohydrate component of an antigen to periodate treatment [59] has been beneficial in interpreting our results. In particular, treatment of *Pcc *antigens demonstrated that cross-reactive nematode-induced IgG3 responses were largely attributed to the carbohydrate component. Interestingly, periodate treatment also reduced apparent cross-reactivity by exposure of protein epitopes, previously masked by carbohydrate, which enhanced the detection of antibodies from mice that had been exposed to the antigen during infection (e.g., anti-pRBC in Fig 3Bii). This allowed us to conclude that levels of anti-pRBC IgG2a antibody in *Pcc-Nb *mice are solely induced by the *Pcc *parasite. Further to this, detection of cross-reactive protein-specific antibodies enabled responses, previously indistinguishable in magnitude between singly-infected groups, to be differentiated. For example, periodate treatment of NbA enhanced detection of *Pcc *induced cross-reactive IgG2a antibodies (X_8 _in Fig 3Biii) whilst the antigen-specific response of *Nb *mice was ablated. This indicates that the level of *Nb*-specific IgG2a observed in *Pcc-Nb *mice is due to *Pcc *driving a cross-reactive IgG2a Th1 type response.

We have demonstrated that the use of serial dilutions and periodate treatment of the parasite antigens can help overcome cross-reactivity for the purposes of analysing and interpreting Th1/Th2 cell immune bias. However, some 'true' cross-reactivity remained (i.e. *Pcc*-induced IgG2a responses to NbA (Fig 2Biii/Fig 3Biii)), and the induction of these antibodies has important implications with regard to biological function. For example the immune responses to nematode infection are typically characterised by a Th2 type (IgG1) response, as we observed for *Nb*-induced responses to the nematode antigen (NbA). The propensity for *Pcc *mice to induce atypical IgG2a antibody isotypes to nematode antigen is likely due to the malaria parasite promoting Th1 cytokines in the environment where the antibody response is established [45]. The biological consequences of the *Pcc *driven IgG2a response to the nematode antigen and the less pronounced IgG1 response of *Nb *mice to *Pcc *antigens remain to be investigated. In *Trichuris muris *infection, manipulation of the immune environment to a Th1 type setting, characterised by elevated IgG2a and IFNγ, was shown to enhance chronicity of this intestinal helminth [62]. *Pcc*-induced IgG2a to nematode antigens may thus have real consequences in terms of disease outcome. Effects of nematode co-infection on the malaria parasite are also evident; *Pcc-Nb *mice have reduced levels of malaria parasitemia in comparison to *Pcc *mice [53] and it is interesting to consider the possibility that cross-reactive IgG2a antibodies induced by the nematode infection may act in concert with the antigen-specific response to control malaria parasites. The potential for cross-reactive responses to have a functional role during infection raises the intriguing possibility that their production is a deliberate strategy of the host to combat diverse parasites [63]. To fully understand the relative contribution of cross-reactive antibodies in parasite control would require passive antibody transfer experiments.

Although schistosome parasites are phylogenetically distinct from nematodes, helminth co-infection studies that investigate *Schistosoma mansoni *provide evidence that cross-reactivity is relevant in other co-infection systems and can have a strong impact on disease severity. Naus et al [43] report the induction of cross-reactive IgG3 antibodies that recognise both *Plasmodium falciparum *and *S. mansoni *antigens. Pierrot et al extended this study, identifying the *S. mansoni *antigen (SmLRR) that is recognised by both malaria and *S. mansoni *singly-infected hosts. Interestingly, as we observed in our *Pcc*-*Nb *model of co-infection, the two infections induce different antibody isotypes to antigen: cross-reactive malaria driven IgG3 and helminth driven IgG4 [45]. In areas co-endemic for these two parasites, exposure to malaria and subsequent induction of the cross-reactive IgG3 response seems to increase the risk of developing hepatosplenomegaly in schistosome infected individuals [44].

## Conclusions

In summary, our data illustrate that whilst cross-reactivity may confound observations of interest, it can largely be overcome by a combination of increasing sera dilution and pre-treatment of antigens with periodate. Adopting such strategies will enable antibody isotypes to be used as an indicator of cytokine bias and clarify interpretation of when Th1-Th2 cell shifts have occurred as a result of co-infection. Arising from this analysis is the opportunity to dissect antigen-specific from cross-reactive responses and thus obtain information pertaining to the relative strength of these responses and recognition of carbohydrate versus protein epitopes. This will provide the foundation on which to base more detailed characterisation of the antibody responses during co-infection in order to investigate their functionality.

## Abbreviations

*Ls*: (*Litomosoides sigmodontis*); *Nb*: (*Nippostrongylus brasiliensis*); *Pcc*: (*Plasmodium chabaudi chabaudi*); LsA: (*Litomosoides sigmodontis *crude antigen); NbA: (*Nippostrongylus brasiliensis *crude antigen); pRBC: (*Pcc *parasitized red blood cell crude antigen); MSP-1_19_: (Merozoite Surface Protein -1_19_); RBC: (Red Blood Cell); Th: (T helper); pi: (post-infection).

## Authors' contributions

KFC participated in the design of the study, conducted the *Pcc-Nb *experiments and immunoassays, performed the statistical analysis and helped to draft the manuscript. TL conducted the *Pcc-Ls *experiments and immunoassays. JL provided recombinant MSP-1_19_. ALG conceived of the study and participated in its design, was involved in all co-infection experiments and helped to draft the manuscript. JEA also conceived of the study, participated in its design and helped to draft the manuscript. All authors read and approved the manuscript.
